# Effects of Objective and Subjective Health Literacy on Patients’ Accurate Judgment of Health Information and Decision-Making Ability: Survey Study

**DOI:** 10.2196/20457

**Published:** 2021-01-21

**Authors:** Peter Johannes Schulz, Annalisa Pessina, Uwe Hartung, Serena Petrocchi

**Affiliations:** 1 Institute of Communication and Health Università della Svizzera italiana Lugano Switzerland; 2 Università della Svizzera italiana Lugano Switzerland

**Keywords:** health literacy, Newest Vital Sign, eHealth Literacy Scale, self-reported health literacy, perception-based health literacy, objective health literacy, performance-based, depression, mental health

## Abstract

**Background:**

Interpreting health information and acquiring health knowledge have become more important with the accumulation of scientific medical knowledge and ideals of patient autonomy. Health literacy and its tremendous success as a concept can be considered an admission that not all is well in the distribution of health knowledge. The internet makes health information much more easily accessible than ever, but it introduces its own problems, of which health disinformation is a major one.

**Objective:**

The objective of this study was to determine whether objective and subjective health literacy are independent concepts and to test which of the two was associated more strongly with accurate judgments of the quality of a medical website and with behavioral intentions beneficial to health.

**Methods:**

A survey on depression and its treatments was conducted online (n=362). The Newest Vital Sign was employed to measure objective, performance-based health literacy, and the eHealth Literacy Scale was used to measure subjective, perception-based health literacy. Correlations, comparisons of means, linear and binary logistic regression, and mediation models were used to determine the associations.

**Results:**

Objective and subjective health literacy were weakly associated with one another (*r*=0.06, *P*=.24). High objective health literacy levels were associated with an inclination to behave in ways that are beneficial to one’s own or others’ health (Exp[B]=2.068, *P*=.004) and an ability to recognize low-quality online sources of health information (β=–.4698, *P*=.005). The recognition also improved participants’ choice of treatment (β=–.3345, *P*<.001). Objective health literacy helped people to recognize misinformation on health websites and improved their judgment on their treatment for depression.

**Conclusions:**

Self-reported, perception-based health literacy should be treated as a separate concept from objective, performance-based health literacy. Only objective health literacy appears to have the potential to prevent people from becoming victims of health disinformation.

## Introduction

### Background

Patients’ knowledge of health matters and their capacity to acquire such knowledge have recently become more important than ever [1]. This has to do with the immense accumulation of health knowledge in medical science and, consequently, the medical profession since the enlightenment. More recently than that, a new ideal emerged, which was to replace older paternalistic conceptions of the relationship between physicians and their patients [2,3]. In the earlier concept, well-meaning physicians based their decisions on their far superior medical knowledge, experience, physical examination, and understanding of what their patients conveyed about their symptoms [3]. Based on this information, physicians developed a diagnosis and prescribed medication accordingly. In the new ideal of patient autonomy, physicians make suggestions to patients, and patients choose which suggestions to follow [3]. Other autonomy concepts include the expert patient, shared decision making, and patient-centered health care [4].

Patient autonomy requires that patients are knowledgeable, which is another reason why knowledge and knowledge acquisition in the field of health are so important today. In addition to knowledge, functional reading and writing abilities, adequate information processing, critical thinking skills, and the capability to make decisions that are beneficial to one’s health [5] are all elements of health literacy. Health literacy is an immensely successful concept in health communication and public health [6]. Its success may be due in part to an intuitively felt paradox that it would be absurd indeed to train health care providers for years and produce expensive pharmaceuticals and high-tech machinery only to witness that patient outcomes are not as good as they could be because patients do not understand what their physician says and physicians fail to notice patients’ lack of understanding [4]. In a sense, the existence of the term “health literacy” is the admission that not all is well with the distribution of medical knowledge in contemporary societies. Not everyone is knowledgeable enough to meet the requirements of the ideal of patient autonomy or capable enough to participate in the communication processes demanded by health care today [7].

When health literacy was first conceptualized, nobody could have known how digital communication would soon change health communication. Due to its extraordinary potential of overcoming traditional limitations to information finding, the internet is today a primary focus of health literacy research [8]. In some respects, it provides solutions or answers to problems associated with the distribution of health knowledge and the spread of health information.

In spite of the many opportunities the internet offers in providing information, social support, conversation exchange, and more advice on prevention than one could ever use, it has also magnified the quality issue in medical care for laypersons who have contact with it. The quality of the information found online is sometimes deplorable [9], and that creates its own problems. For individuals and organizations, advocating the potential benefits offered by information technology is fairly frustrating. A new, two-sided medium was introduced, which offered a way to seek and find health information more or less everywhere, at any time, on every subject, and at a very low cost. However, what people have put on the medium is, to a considerable degree, medical disinformation.

Fortunately, a solution was at hand: health literacy. If health literacy were to include the ability to discriminate true and reliable health information websites from fraudulent and misleading ones, information seekers online could learn which sources provide high- or low-quality information and could base their conclusions about the quality, and hence the credibility, of specific information on the general quality of the source. Individuals who did not have adequate health literacy could receive training to increase their health literacy or physicians could talk to them on the patient’s level. This raises the question of whether individuals with a high level of health literacy also have the capability of appraising the general quality of digital sources of health information.

The hope is that when people with adequate online health literacy encounter low-quality health information, they will recognize it for what it is, and when a website is of low quality and the patient knows it, they will not accept its content and not change their stored knowledge of the subject. Some might even scan their existing knowledge when they meet a website with health disinformation and erase the knowledge that is associated with the problematic website newly encountered. That is to say, people with high health literacy might indeed be protected against the influence of disinformation and, in favorable conditions, even improve their knowledge. In contrast, people with inadequate eHealth literacy will not recognize the low quality, accept its content, and store it as newly gained knowledge of the subject.

Although people’s awareness of their health literacy has not yet been considered, it should be. People might perceive themselves as more health literate than they actually are, and they might act on that misperception. It is obvious that an exaggerated misperception of one’s own health literacy cannot be a good advisor for medical judgment, decisions, and behaviors. Earlier publications referred to these persons as “dangerous self-managers” [10]. Misperception of one’s self is a rather well-known phenomenon in social psychology, where it exists under labels such as optimistic bias, third-person perception, and looking-glass perception.

Although health literacy is conceptualized as an objective feature, it has increasingly been measured subjectively—that is, as a self-perception—in academic research [11]. Measures of health literacy based on self-perceptions are often problematic because people might deceive themselves about their health literacy abilities [12]. At the same time, such measures gain ground in research. One example is the Brief Health Literacy Screen (BHLS), which asks the respondent 3 questions about their confidence in understanding medical information when they seek help from health care institutions [13]. Another example is the eHealth Literacy Scale (eHEALS), which consists of 8 items measuring consumers’ knowledge, comfort, and perceived skills as they relate to finding, evaluating, and applying electronic health information to health problems [14]. The final example is the European Health Literacy Survey Questionnaire (HLS-EU-Q47), which assesses how easy or difficult the patient perceives health-related tasks to be [15].

Most commonly, the objective, performance-based measures require individuals to demonstrate their knowledge and familiarity regarding medical terms (eg, Rapid Estimate of Adult Literacy in Medicine [REALM] [16]) or their understanding of sentences by their ability to fill in gaps within them (eg, Test of Functional Health Literacy in Adults [TOFHLA] [17]), or to complete tasks demanding numerical ability (TOFHLA [17], STAT-interest and STAT-confidence scales [18], and Lipkus Numeracy Scale [19]). Self-reported measures of health literacy involve individuals describing themselves and their skills. Self-reported measures target the individuals’ self-perceived ability to find information and understand it, and to assess individuals’ confidence in their own health literacy. Subjective measures of objective concepts have long been used in survey research, often for concepts that could be openly labeled, such as knowledge or intelligence [20]. Subjective impressions of how much of these qualities individuals think they have can be ascertained directly because the concepts have spilled over into normal, nonacademic language. That has not yet happened to the concept of health literacy. Inquiry into individuals’ subjective perception of their health literacy must, therefore, be performed indirectly and include questions about behaviors and processes that indicate health literacy. Self-reported measures are much more susceptible to individual and cultural influences, such as social desirability and beliefs about health and illness [21]. Conversely, a performance-based measure assumes that it is unlikely that someone would purposefully cheat on it by intentionally appearing less capable than they are [21].

The validity of a self-perceived indirect measure of health literacy can, therefore, be judged by comparing its results with the results of performance-based instruments.

Self-report and performance-based measures of health literacy have been extensively investigated in their ability to predict a range of health-related behaviors. One study [22], for example, demonstrated that performance-based numeracy and literacy, considered together, predicted skills to perform health tasks among older adults. Along the same lines, Gazmararian et al [23] showed that patients with inadequate performance-based health literacy were less likely to adhere to their physicians’ treatment a year later. People with limited health literacy were less likely to have sought cancer information and more frequently endorsed fatalistic beliefs about their decision making [24].

On the other hand, even low levels of self-reported health literacy seem to be associated with worse health outcomes. One study in patients with diabetes [25] found that low self-reported health literacy was associated with less diabetes knowledge, glycemic control, and physical activity. Mitsutake et al [26] found mixed results. Individuals with high self-reported health literacy were significantly more likely to exhibit good health behaviors, perform physical exercise, and eat a balanced diet. Still, there were no associations of health literacy with cigarette smoking, alcohol consumption, hours of sleep, or eating between meals. Wångdahl et al [27] found that refugees with limited self-reported health literacy reported worse health, impaired well-being, and fewer health care–seeking behaviors than those with high health literacy.

A systematic review [11] found a paucity of research analyzing the relationship between objective and subjective health literacy and health outcomes. Haun et al [28] found no significant relationship between limited health literacy and patient self-report of having or not having hypertension, diabetes, or a past stroke after adjusting for covariates (ie, gender, race, education, self-reported reading level, retiree status, and having a functional disability). Hirsh et al [29] found a significant association between the self-reported measure of health literacy and a multidimensional measure of health, which did not hold with a performance-based tool. Kiechle et al [11] found no differences in the relationship between performance-based and self-reported health literacy for 4 of 6 outcomes (self-reported diabetes, stroke, hypertension, and a physician-completed rheumatoid arthritis disease activity score).

### Hypotheses

The broader question that this research addressed was the utility of self-perceived health literacy in health communication research. The considerations outlined above suggest three comparisons that should help to assess this utility: (1) the association of self-perceived and objective health literacy, (2) the influence of health literacy on choice of behavior, and (3) the ability to recognize disinformation on the web. These variables were seldom studied together and the results were inconsistent [30-36]. In a unique study by Benotsch et al [37], they found an overall positive association between low health literacy levels and wrong perceptions of information quality. Specifically, low health literacy was associated with lower quality ratings of a high-quality website and higher quality ratings of a low-quality website.

Therefore, our primary research question (RQ1) was as follows:

- RQ1: How strongly will a self-perceived measure of health literacy be associated with a performance-based measure?

Four hypotheses were formulated to present the associations in a testable format:

- H1: Participants who score high on an objective, performance-based test of health literacy will make better treatment choices for depression than participants who score low.
- H2: Participants who score high on an objective, performance-based test of health literacy will make better judgments on the quality of mental health websites.
- H3: Participants who score high on an objective, performance-based test of health literacy will make better treatment choices based on their judgments of the quality of mental health websites, reflecting a mediation of the relationship between health literacy and depression-related decision making by the perception of website quality.
- H4: Self-reported health literacy will not produce similar associations.

## Methods

### Study Design

The study is part of a larger experiment testing an aspect of the order of persuasive communication content. This experiment and the order of presentation are of no concern to this paper. Therefore, we did not include order as a variable here.

The experimental design, however, had consequences for the structure of the data collection. We considered it important for the experiment to have roughly equally sized groups of high and low objective health literacy. For this purpose, data were collected in two steps. The first step involved an assessment of objective health literacy and the collection of sociodemographic information. The findings of the first step were used to define high and low literacy and to assign participants into experimental groups randomly. The second step consisted of all other measures. Two pilot studies were carried out before the study was conducted (see Multimedia Appendix 1).

### Variables and Measures

The independent variables were two measures of health literacy: (1) a subjective, perception-based measure, and (2) an objective, performance-based measure. The measures were available as total scores or as dichotomous summaries (low/inadequate versus high/adequate level). An intervening variable was the perception of the quality of two websites, one of dubious quality, the other of high quality. The dependent variable was treatment choice, considered as a dichotomous variable. Several covariates were also considered.

Performance-based health literacy was measured using the Newest Vital Sign (NVS) [38], which uses a fictitious ice cream label. It measures literacy, comprehension, numeracy, application/function, and evaluation skills by asking questions that are answered after a person observes the label. The final score ranges from 0 (limited literacy) to 6 (adequate literacy); the dichotomy separated participants with low/inadequate health literacy (scores of 0-4) from those with high/adequate health literacy (scores of 5-6). The scale demonstrated good internal reliability (α=.62).

Self-reported health literacy was measured using the eHealth Literacy Scale (eHEALS) [14,39]. Online health literacy, or eHealth literacy, refers to “the ability to seek out, find, evaluate and appraise, integrate, and apply what is gained in electronic environments toward solving a health problem” [40]. The scale is an 8-item, self-reported measure developed to assess consumers’ combined knowledge, comfort, and perceived skills at finding, evaluating, and applying eHealth information to health problems. Responses are measured using 5-point Likert scales whose options range from 1 (“strongly disagree”) to 5 (“strongly agree”). The total scores were calculated as the mean, with the ratings formatted such that higher scores (ie, closer to 5) represented higher self-reported eHealth literacy. The scale demonstrated good internal reliability (α=.89) and internal consistency (r_s_>0.58; mean 3.17, SD 0.78). The dichotomous form was computed based on the median split (median 3.25), thus generating one group with low (n=178) and one group with high (n=184) self-reported health literacy.

#### Website Quality

Two versions of a mock website informing about treatment options for depression were produced, one of high quality and the other of low quality. To provide our study with ecological validity, we retrieved the contents from real websites, one of high quality [41] and the other of low quality [42]. Both appeared among the top results on Google.it when the keywords “anti-depressive” and “natural remedies for depression” were searched. The criteria that we used to identify a high-quality website were having a main focus on the most effective treatment options according to scientific research (namely antidepressant medication and psychotherapy), and a clear debiasing intention toward the most popular misconceptions about them [43]. Conversely, the criterion for low-quality was the emphasis on common misbeliefs about treatment [43], including the overestimation of the curative effect of natural and self-help remedies [44]. However, in contrast with the real websites, the amount of information on the mock websites was shortened in order to make the stimuli comparable in terms of number and pieces of information provided, number of words, and overall cognitive effort required. Moreover, we used the same layout for both stimuli (eg, color, graphics) and a simple screenshot instead of an interactive website in order to limit the effects due to uncontrolled variables (see Multimedia Appendix 2). A 1-item manipulation check was included to identify if people had read, understood, and could recall the information on the website.

#### Perceived Information Quality

Participants’ perceptions of information quality were assessed using 7-step semantic differential scales [30,45]. Positive adjectives were “accurate,” “reliable,” “complete,” and “understandable,” while the respective opposites were “inaccurate,” “unreliable,” “incomplete,” and “nonunderstandable.” High numbers on the scales indicated positive perceptions. The data showed good internal reliability (α=.88 for high-quality websites, and α=.90 for low-quality websites) and internal consistency (r_s_>0.68 for high-quality websites, and r_s_>0.75 after removing the item understandability). Consequently, the scores for reliability, accuracy, and completeness were considered in the analyses.

#### Outcome Variable

From a list, participants were asked to choose one or more treatments for depression for themselves (help-seeking behavior), a family member, and a close friend (advice-giving behavior). The Kuder-Richardson 20 coefficient indicated good internal consistency among the three items (KR20=0.96), thus providing the basis to develop a single measure of the construct. The final score ranged from 1 (correct choice) to 0 (wrong choice). Examples of the right choices were psychotherapy, antidepressant medications, and seeking help from a doctor. Examples of wrong decisions were treating depression with St John’s wort or vitamins or yoga without any mention of antidepressant medications or psychotherapy.

#### Covariates

The frequency of online health-related information–seeking behavior in general as well as about depression in particular were both asked with a single item. The response options ranged from 1=never to 6=more than two times a week. During the scoring procedure, both items were reversed, with higher scores indicating lower frequency. Participants’ previous experience with depression was assessed in terms of having ever suffered from depression or helping someone close to them, such as a family member or a close friend, who was suffering from depression. The response options were “no previous experience” or “yes previous experience.”

### Sample

The study was conducted in Italy using a snowball sampling method by posting the link to the survey on several public and private Facebook pages. The survey was implemented on QualtricsXM software (version 2019; Qualtrics). The inclusion criteria for study participation were adults aged between 18 and 65 years who were residing in Italy, with a good command of the Italian language and internet access. Mental health workers (psychiatrists, psychologists, and psychotherapists) and psychology graduates/students were excluded from the sample.

An a priori power analysis was conducted using G*Power 3.1.9.4 software [46] to determine the sample size (with α=.05, power=.95, η^2^=0.05), and the final estimate was 331 participants.

A total of 501 participants completed the first part of the survey (ie, performance-based health literacy and sociodemographics). Among them, 380 participants completed the second part of the survey and 121 participants dropped out. Of the 380 participants, 18 were excluded because their answers to the postmanipulation check were incorrect, suggesting deficiencies in their perception of the website being assessed. Comparisons between dropouts and participants who completed the surveys did not show any significant difference in terms of gender, age, educational level, or performance-based health literacy. The final number of participants was 362. The subjects were aged between 18 and 66 years (mean age 35.54 years, SD 13.76 years) and 72.1% (261/362) were female. The majority of the participants had a high school degree (145/362, 40.1%), a bachelor’s degree (73/362, 20.2%), or a master’s degree (134/362, 37.0%).

### Statistical Analysis

SPSS Statistics for Windows (version 25.0; IBM Corp) and Hayes’ PROCESS macro v3.4 for SPSS 25.0 [47] were applied. The internal reliability and consistency of the continuous scales were calculated using α and r_s_, and the Kuder-Richardson 20 coefficient was used for noncontinuous scales. Normality distribution was found with skewness values ranging between –1.1 and 0.44 and kurtosis values ranging between 0.9 and –0.62, except for the variable assessing the information-seeking behavior about depression (skewness 2.5, kurtosis 6.6). Chi-square tests and *t* tests were calculated to compare the subsample of participants who completed only the first part of the survey with those who completed the entire survey. Pearson correlations were calculated between continuous variables and point-biserial correlation coefficients (*r*_pb_) were calculated between binary and continuous variables. We conducted *t* tests to compare the mean differences in perceived information quality and the level of health literacy. Logistic regression was performed with treatment choice as the outcome variable and gender, age, past experience with depression, both measures of health literacy, and an interaction term between health literacy and perception of website quality as independent variables. Using the entry method, we ran a three-step model, including the relevant sociodemographic variables first, then the possible predictors of the main effects on treatment choice, and lastly the interaction term. Finally, mediation analyses were carried out on perceived information quality as a mediator between health literacy and treatment choice.

## Results

### Bivariate Findings

The two measures of health literacy were not associated (*r*=0.06, *P*=.24, n=362). In response to RQ1, it can be said that the correlation shows that there is little reason to hold that the two scales measure the same thing.

Correlation analysis also showed that respondents with higher (as compared with lower) levels of performance-based health literacy perceived a low-quality website as more negative and were inclined to choose beneficial behaviors for themselves, family, and friends. In contrast, when respondents were grouped according to perception-based health literacy, no such difference appeared. However, those with high health literacy levels rated a high-quality website as more positive than those with low levels did. This supports H1 and also H2, but only for the judgment of low-quality websites. It also supports H4, except for positive websites, which were perceived more positively by individuals who perceived themselves as highly health literate and for whom no such association was expected. Correlations between health literacies and dependent variables are displayed in Table 1.

**Table 1:** Correlations between two health literacy measures and perception of website quality/choice of depression treatment.a

	Objective, performance-based measure (NVS)^b^, n=362		Subjective, perception-based measure (eHEALS)^c^, n=362	
	*r*	*P* value	*r*	*P* value
Perception of high-quality website	0.02	.78	0.14	.009
Perception of low-quality website	–0.14	.006	–0.08	.13
Treatment preferences	0.12	.007	0.01	.81

^a^High numbers indicate high health literacy, positive perception, and beneficial preferences for treatment.

^b^NVS: Newest Vital Sign.

^c^eHEALS: eHealth Literacy Scale.

An independent samples *t* test was conducted to evaluate whether the performance-based health literacy level (low versus high) had an effect on the perceived information quality of the low-quality website. The analysis was significant (*t*(360)=2.81, *P*=.005), with participants who had higher performance-based health literacy achieving lower scores (mean 3.70, SD 1.53) than participants who had lower performance-based health literacy (mean 4.17, SD 1.61). The same analysis was conducted considering self-reported health literacy, but the result was not significant (*t*(360)=1.064, *P*=.29).

In the next step, we tried to build linear regression models for predicting the perception of high- and low-quality mental health websites and treatment choice. We expected respondents with high levels of objective health literacy to evaluate the two websites more consistently than respondents with low levels of health literacy and make a better treatment choice. Consistent evaluation means that the high-quality and low-quality websites were assessed positively and negatively, respectively. A similar difference was not expected to emerge for respondents with different levels of subjective health literacy. Predictors were entered stepwise: the sociodemographic variables (gender, age, education) were entered first, then the covariates indicating experience, and finally the measures of health literacy. Table 2 shows the coefficients of the regression analyses.

**Table 2:** Regression analyses for predicting the perception of high- and low-quality mental health websites and treatment choice.

Step		Evaluation of high-quality website (linear regression)		Evaluation of low-quality website (linear regression)		Treatment choice (binary logistic regression)	
		β	*P* value	β	*P* value	Exp(B)	*P* value
**Step 1: Sociodemographics**							
	Gender (0=males)	–.056	.30	.035	.497	1.866	.02
	Age	.002	.98	.122	.02	0.990	.29
	Education level (0=low)	–.036	.50	–.179	<.001	0.998	.99
	*R*^2^ (%)	0.3		4.1		4.8	
**Step 2: Experience**							
	Health information–seeking	.004	.95	.028	.63	1.042	.77
	Depression-related information–seeking	.023	.69	–.052	.36	1.108	.31
	Past experience of depression	–.018	.75	–.128	.02	1.306	.30
	*R*^2^ (%)	0.7		5.1		6.2	
**Step 3: Health literacy**							
	Objective, performance-based health literacy (NVS^a^)	–.019	.73	–.099	.07	2.068	.004
	Subjective, perception-based health literacy (eHEALS^b^)	.144	.01	–.041	.47	0.697	.04
	*R*^2^ (%)	2.4		5.6		10.4	
**Step 4: Perception of mental health websites**							
	High-quality site	N/A^c^	N/A	N/A	N/A	1.253	.03
	Low-quality site	N/A	N/A	N/A	N/A	1.088	.74
	*R*^2^ (%)	N/A		N/A		18.6	
**Step 5:** **Interaction**							
Interaction between objective health literacy and perception of a low-quality site		N/A		N/A		0.886	.035
*R*^2^ (%)		N/A		N/A		20.1	

^a^NVS: Newest Vital Sign.

^b^eHEALS: eHealth Literacy Scale.

^c^N/A: not applicable.

The first noteworthy result was that the only predictor of a favorable perception of a high-quality website was self-perceived health literacy. A low-quality website was evaluated negatively by highly educated persons, by persons with past experience of depression, and almost (missing significance by a small margin) by objective health literacy. Treatment choice was influenced by gender, as well as by objective health literacy (beneficially) and subjective health literacy (detrimentally). Recognition of a high-quality website by positive perception also increased participants’ likelihood of making a sound treatment choice.

The interaction analysis showed that individuals with a high level of objective health literacy and an accurate recognition of a low-quality website as being problematic exercised good judgment regarding treatment behaviors. In particular, participants with high performance-based health literacy were more likely to choose the right treatment for depression than participants with low performance-based health literacy.

**Figure 1:**
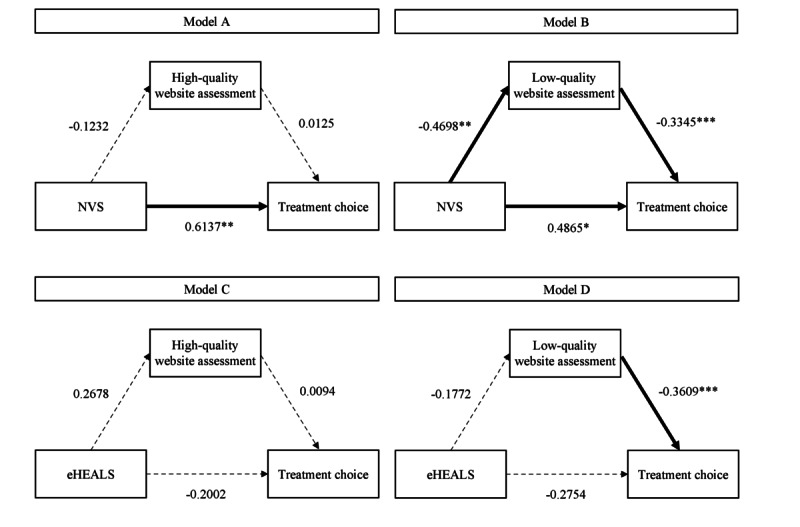
Mediation analyses. eHEALS: eHealth Literacy Scale; NVS: Newest Vital Sign. *P<.05; **P<.01; ***P<.001.

The mediation analysis yielded similar results. First, an overall path led from health literacy to treatment choice, but only when health literacy was operationalized as NVS—that is, an objective, performance-based measure. The subjective measure showed no relationship between health literacy and treatment choice. Second, when health literacy was operationalized objectively and respondents were given a low-quality website to assess, they recognized the low-quality website for what it is, and that recognition improved their judgment of treatment alternatives. Third, individuals with high levels of subjective health literacy did not have better insight into the quality of websites and did not assess website qualities differently than individuals with lower health literacy; however, if they recognize the poor quality of the website, they are also capable of opting for better treatments. In other words, individuals with high levels of subjective health literacy were prone to overlook the poor quality of web content, but if they do not overlook it, they will make better treatment decisions (Table 3 and Figure 1).

**Table 3:** Results of mediation analysis.

Mediation analysis		Model A	Model B	Model C	Model D
**Model specification**					
	Health literacy indicator	NVS^a^	NVS	eHEALS^b^	eHEALS
	Quality of website to be assessed	High	Low	High	Low
**Model**					
	Path from health literacy to website assessment	–0.1232 (*P*=.38)	–0.4698 (*P*=.005)^c^	0.2678 (*P*=.05)	–0.1772 (*P*=.28)
	Path from health literacy to treatment choice	0.6137 (*P*=.007)^c^	0.4865 (*P*=.04)^c^	–0.2002 (*P*=.37)	–0.2754 (*P*=.23)
	Path from website assess­ment to treatment choice	0.0125 (*P*=.88)	–0.3345 (*P*<.001)^c^	0.0094 (*P*=.91)	–0.3609 (*P*<.001)^c^
**Effect**					
	Direct effect of health literacy on treatment choice	0.6137 (*P*=.007)^c^	0.4865 (*P*=.04)^c^	–0.2002 (*P*=.37)	0.2754 (*P*=.23)
	Indirect effect of health literacy on treatment choice	–0.0015 (*P*=.92)	0.1576 (*P*=.02)^c^	0.0025 (*P*=.92)	0.0640 (*P*=.31)
**Model summary**					
	–2LL	455.2257	434.8030	462.8626	437.7285
	*P* value	.02	<.001	.67	<.001

^a^NVS: Newest Vital Sign.

^b^eHEALS: eHealth Literacy Scale.

^c^Coefficients with *P*<.05.

## Discussion

### Association of Performance-Based and Perception-Based Hypotheses (RQ1)

For a summary of the results, we feel that the research question highlights the necessity to differentiate subjective and objective health literacy more clearly, conceptionally as well as in terms of measurement. One finding to support this view is the weak association that was found between the two kinds of information. Another reason to differentiate them is because of the different influences that the two kinds of health literacy had on the study outcomes—the assessment of the quality of internet information (as an intermediate variable) and the choice of treatment behaviors, in line with official recommendations.

### Effect on Outcomes (H1 and H4)

In general terms, the answer to RQ1 was that performance-based and perception-based health literacies were uncorrelated, and they played very different parts in predicting the dependent variables. These results and other published research [10] suggest that we need to think seriously about referring to apparently very different concepts by the same name. The study clearly shows that health literacy positively affects patients’ choice of treatment, “positively” indicating that high health literacy and beneficial decision making are linked. However, this was true only for objective, performance-based health literacy and not for subjective, perception-based concepts of health literacy. This clearly supports H1 and H4, and it means that the perception-based version of health literacy is lacking the major asset of the concept—improved judgment regarding medical treatments.

### Role of Intermediate Variables (H2 to H4)

Health literacy helps patients to identify low-quality health information on the web (H2), and those who recognize websites of low quality tend to make better treatment choices (H3). Both assertions are true for patients whose health literacy is defined objectively; when it is defined subjectively, the link between literacy and quality perception disappears (H4), although the effect of recognizing the problematic site remains. In other words, patients who recognize a low-quality website have better judgment, but a subjective classification of health literacy does not play a role in that recognition.

The objective, factual ability to find one’s way through the communicative maze of present-day health care has several components, including the ability to describe one’s symptoms to the doctor and understand the doctor’s reasoning of diagnosis and treatment. It is something genuinely different from patients’ personal impressions that a consultation went well, that they succeeded in presenting their case, and that they understood what the doctor had to say [7].

Some but not all expectations about performance-based health literacy were met in this study. Performance-based literacy predicts the recognition of the shortcomings of low-quality mental-health websites (H2) and good judgment with regard to treatment (H1), and health literacy and accurate perceptions together predict good treatment choices even better (H3). Favorable evaluations of websites, in contrast, seem unrelated to this kind of health literacy, other than what was hypothesized in H2, which pertained to both low- and high-quality websites. Although perception-based health literacy was associated with favorable perceptions of high-quality websites, which went against H4, H4 was otherwise supported.

In the wake of this analysis, health literacy no longer appears to provide the certain benefits that we initially described. However, objective health literacy can still be perceived to be a valuable asset. Its association with sound judgments on treatment and its relationship with negative evaluations of low-quality websites reflect its value. After reviewing our results, the same value cannot be placed on subjective, perception-based health literacy, which might fail to lead to a successful result even if it does not actually do harm.

Actions aimed at enhancing health literacy should not stop at influencing subjective health literacy. The more important point of attack seems to be objective health literacy, and leaving it out of the picture runs the risk of failing to reach the desired outcome. The autonomy policies in health care today are an example of this. They might easily induce in patients a desire to make treatment choices by themselves, and with this growing desire, patients may increasingly consider themselves to be the agency in charge; this might blind individual patients to the fact that nothing much has changed with regard to the objective abilities they possess.

Health literacy on one side and autonomy ideals on the other side complement one another. The abilities that come with health literacy can be used to prevent patients from making too many detrimental decisions, and the autonomy granted would give their health literacy a raison d’être as it would provide a chance to apply their newly acquired ability in the real world, under real circumstances and with real consequences. It has to be noted, though, that health literacy is understood as being of assistance to physicians, not as their replacement.

The desire to counter internet disinformation by increasing health literacy is based on patients’ ability to recognize the low quality of some of the messages on the web. According to our results, this can work for objective, performance-based health literacy, but not for the subjective version of the concept. This might be explained by a specific shortcoming of thinking: one is relatively satisfied with their communication abilities because one cannot imagine how much better they could be. This would apply to the ease with which one communicates in health contexts as well as to the quality of websites. Low standards, applied to oneself and to websites as well, could be the common problem behind the measured association.

We expected to find a differential effect on an individual’s decision making based on what they know and what they think they know on the given topic [5,10]. In this vein, we expected that self-reported health literacy (ie, what individuals thought they knew) would not make any difference in the depression-related decision making compared with performance-based health literacy (ie, what individuals actually knew). The hypothesis was confirmed because we found that high objective health literacy predicted sound treatment choice, whereas high subjective health literacy did not. Measuring concepts as subjective entities, as is done for intelligence and knowledge [20], goes back to the idea that people react primarily not to objective states but to their perception of those states. Asking people what they think they know, or how intelligent they think they are, works well because these are everyday terms. Health literacy, however, is not an everyday concept, and therefore its measurement must be performed indirectly.

Moreover, subjective and objective health literacy did not correlate. This result was in line with the literature supporting the inadequacy of self-reported measures to detect one’s actual health literacy level [48] and demonstrated the fact that the two measures evaluate different constructs [21,39,48,49].

We also tested whether information quality mediated the relation between health literacy and depression-related decision making, first considering performance-based and then self-reported health literacy. The analyses revealed that the perceived quality of a low-quality message partially mediated the relation between the performance-based health literacy level and the treatment choice. Therefore, the performance-based health literacy level not only had a positive direct effect on treatment choice but it also had a positive effect on treatment choice through its effect on the perceived quality in the case of a low-quality message. In other words, the higher the actual health literacy level, the lower the perceived quality of a low-quality website, and the better the treatment choice, while the lower the actual health literacy, the higher the perceived quality of a low-quality message, and the worse the decision making. This finding is meaningful because it suggests that efforts to increase the objective health literacy level have the potential to improve not only the judgment of information quality but also the appropriateness of the decision making.

Moreover, in the various mediation models examined, the main effect of performance-based health literacy on treatment choice was always significant (H1), while the main effect of self-reported health literacy was not (in any of the cases [H4]). This result suggests that the self-reported health literacy level does not have the potential to improve the quality of judgments and decisions.

To sum up, believing oneself to be health literate does not imply that one is able to make accurate perceptions of the quality of health information presented to them, nor does it mean that one is able to make good health-related decisions. Feeling empowered, indeed, was argued not to be a sufficient requisite for making good decisions [5,10]. These findings provide further support to the hypothesis that a difference actually exists between performance-based and self-reported measures of health literacy. Interpreting self-confidence as health literacy does not suffice; the more consequential performance-based level of health literacy counts more [21,29,48,49], especially in terms of making accurate judgments of health information.

### Strengths and Limitations

One main limitation of the present study is the generalizability of its results. We recognize that there might have been a selection bias due to the sampling method used; however, a positive aspect was that the sampling was conducted via Facebook. Therefore, we were very likely to sample actual internet users. Another limit to the generalizability of our findings pertains to the ecological validity of the study. Indeed, we selected the mock versions of two actual websites as experimental stimuli. However, in the future, better selection criteria could be established by reviewing the main Italian information websites about depression using appropriate tools, such as DISCERN [50].

Strictly speaking, the analysis cannot claim to say anything beyond the findings on mental health and depression websites and their users. The results were nevertheless formulated without constant reminders of this limitation. This can be interpreted as a hint that we expect to find similar associations for other health topics as well.

### Conclusions

The take-home messages of the present research are as follows. Our findings provide further support to the hypothesis that a difference exists between performance-based and self-reported measures of health literacy. In particular, performance-based health literacy is associated with the ability to recognize faulty information, sound judgment and health-related decision-making skills, and good health outcomes. These are largely the consequences that make health literacy such a cherished concept. On the other hand, individuals who boast of experience that might indicate health literacy (ie, the items in the subjective, self-reported measures) cannot be regarded as having similar abilities, especially in the absence of objective health literacy to support their conception of their own abilities. Simply because someone believes that they communicate well in health care environments does not mean they actually do and no one should assume that they do. The state of not knowing what one does not know has several consequences. First, other individuals in the health care system have to assume that, despite the professed communicative abilities, these individuals are prone to become victims of medical disinformation. Second, some questions arise. For performative measures, research should ask how much health literacy is needed to avoid adverse decisions in health care. For self-reported literacy measures, the poor construct validity for health literacy suggests that these measures need to be tested directly to determine whether they are valid measures of empowerment [10].

In terms of health policies, the conclusion is that patients should not be encouraged to claim a larger share in medical decision making unless it is clear that a sufficient basis of objective health literacy exists.

## Appendix

Multimedia Appendix 1 Pilot studies.

Multimedia Appendix 2 Websites.

## Abbreviations

BHLS: Brief Health Literacy Screen
eHeals: eLiteracy scale
HLS-EU-Q47: European Health Literacy Survey Questionnaire
NVS: Newest Vital Sign
REALM: Rapid Estimate of Adult Literacy in Medicine
TOFHLA: Test of Functional Health Literacy in Adults
