# Supplementary material for: Effects of Objective and Subjective Health Literacy on Patients’ Accurate Judgment of Health Information and Decision-Making Ability: Survey Study
Source: J Med Internet Res. 2021 Jan 21;23(1):e20457. doi: 10.2196/20457 (PMC7861996; doi:10.2196/20457)
Supplement: Multimedia Appendix 1 [file jmir_v23i1e20457_app1.docx]

**Multimedia Appendix 1**

**Pilot Studies**

We conducted two pilot studies before starting data collection: the first one to evaluate the contents of the mock websites (experimental stimuli) and the second one to test the functioning and understandability of the whole survey.

In the first pilot study, four psychologists entirely blind for the study objectives, and hypotheses were asked to evaluate the two websites, named with the generic labels “A” and “B”. Specifically, they were given two tasks: identify the high-quality and low-quality website and provide any open feedback that came to their mind. A similar procedure was followed to assess whether the questions of the post-manipulation check were unmistakable if one had (carefully) read the information provided. All the psychologists correctly identified the stimuli, and, based on their feedback, small changes to the wording were made until consensus was achieved.

In the second pilot study, seven subjects (four males) matching the criteria for study participation were administered both parts of the survey to test the functioning and understandability of the whole instrument developed for data collection. Four out of 7 pilot surveys were conducted in the presence of the experimenter and using the thinking aloud technique to detect eventual difficulties (actual and perceived) during completion. No relevant barriers were found. Participants were also asked the same evaluation of the websites and the questions of the post-manipulation check as the psychologists did in the first pilot, but no further changes were made.

Results of the pilot studies showed that the questionnaires and websites were understandable, not cognitively demanding and that the time taken to complete each part was in line with our estimates.
