# Supplementary figures and images for: Effects of Objective and Subjective Health Literacy on Patients’ Accurate Judgment of Health Information and Decision-Making Ability: Survey Study
Source: J Med Internet Res. 2021 Jan 21;23(1):e20457. doi: 10.2196/20457 (PMC7861996; doi:10.2196/20457)

**Multimedia Appendix 2**


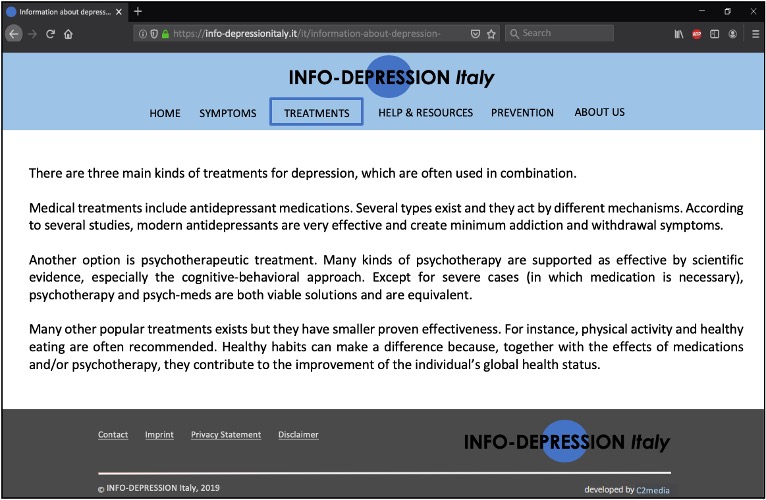

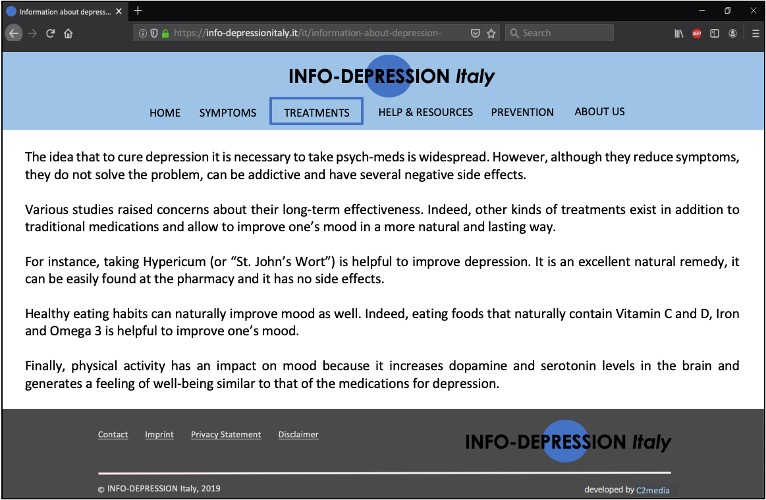
**Websites**

High-quality website:

Low-quality website:

Supplement: Multimedia Appendix 2 [file jmir_v23i1e20457_app2.docx]
